# On the Present State of Medicine in Spain

**Published:** 1836-01

**Authors:** 


ON THE PRESENT STATE OF MEDICINE IN SPAIN.

As it may be some time before we receive the full report on the State of Medicine and the Medical Institutions of Spain, which is in preparation for us by a distinguished physician and scholar of that country, we present our readers, in the meanwhile, with the following short extract of a letter, just received from our medical correspondent at Madrid, and which possesses a melancholy interest, from the illustration it affords of the miserable state of the medical profession in that fine but long-misgoverned country.

"The medical profession in Spain, as regards its present condition, is in complete harmony with every thing else;—that is to say, it is in a state of revolution. Medical men belong, and with few exceptions have always belonged, to the Liberal party; and the class of *pure Physicians* consisted, almost to a man, of warm Constitutionalists, in the year 1820. By the term pure physicians we understand, in Spain, all those who belong to the universities, in contradistinction to those who belong to the colleges of surgery. When the Constitution was overthrown in 1823, the then absolute king, Ferdinand, incensed against the whole body of pure physicians, worked them all sorts of annoyance, and with much success, as you may believe. At last, and with the view of punishing them most effectually, he issued a decree, in which he commanded that no pure physician should be employed in the palace, the hospitals, or in any establishment under government; that is to say, in no public situation whatever, since in Spain all establishments are more or less under the control of the government; and, to supply their places, he converted surgeons into physicians by royal order, commanded that the colleges of surgery should be of medicine also, and created a great many young men at that time in the colleges *Physician-Surgeons*, (Medico-Cirujanos,) as they are called. The pure physicians, thus expelled from the court, hospitals, the establishments for mineral waters, &c., were compelled to bear their wrongs in silence till Ferdinand died. Since that event they have been in open war with the Board of Medicine, the members of which are the same physician-surgeons now to the queen as they were before to Ferdinand. Unfortunately for the pure physicians, the president of the board, one of the physicians made by royal command, enjoys the confidence of the queen, and until lately has been able to resist the attacks, not only of the physicians but even of the Cortes, by whom the edict of Ferdinand has been unanimously condemned. At length, however, in consequence of the late revolution, and consequent change of government, the president has resigned, and there is now a general expectation that the medical profession will be restored to order.

"The present state of Spain is, as you may well believe, very adverse to the cultivation of science and literature: nobody thinks of any thing but politics. Six months ago we had only four medical Journals in all Spain, and at present we have only two! The four Journals were, *La Gaceta Medica* and *El Boletin de Medicina*, at Madrid; *La Biblioteca Medica*, at Zaragoza; and *Los Archivos Homiopaticos*, at Cadiz. The *Gaceta*, the journal of the physician-surgeons, died two months since of inanition; and I believe the *Archivos* shared a similar fate; so that now we have only the *Boletin*, the organ of the pure physicians, and the *Biblioteca*, which has very few subscribers."

*Madrid; Oct.* 3, 1835.

